# Simultaneous Multi-Slice Cardiac MR Multitasking for Motion-Resolved, Non-ECG, Free-Breathing T1–T2 Mapping

**DOI:** 10.3389/fcvm.2022.833257

**Published:** 2022-03-04

**Authors:** Xianglun Mao, Hsu-Lei Lee, Zhehao Hu, Tianle Cao, Fei Han, Sen Ma, Fardad M. Serry, Zhaoyang Fan, Yibin Xie, Debiao Li, Anthony G. Christodoulou

**Affiliations:** ^1^Biomedical Imaging Research Institute, Cedars-Sinai Medical Center, Los Angeles, CA, United States; ^2^Department of Bioengineering, University of California, Los Angeles, Los Angeles, CA, United States; ^3^Department of Radiology, University of Southern California, Los Angeles, CA, United States; ^4^Siemens Medical Solutions, Inc., Los Angeles, CA, United States

**Keywords:** multiparametric magnetic resonance imaging, simultaneous multi slice, cardiovascular imaging, free breathing cardiac MR, non-ECG gated, low rank tensor completion

## Abstract

The aim of this study is to simultaneously quantify T1/T2 across three slices of the left-ventricular myocardium without breath-holds or ECG monitoring, all within a 3 min scan. Radial simultaneous multi-slice (SMS) encoding, self-gating, and image reconstruction was incorporated into the cardiovascular magnetic resonance (CMR) Multitasking framework to simultaneously image three short-axis slices. A T2prep-IR FLASH sequence with two flip angles was designed and implemented to allow B1+-robust T1 and T2 mapping. The proposed Multitasking-SMS method was validated in a standardized phantom and 10 healthy volunteers, comparing T1 and T2 measurements and scan-rescan repeatability against corresponding reference methods in one layer of phantom vials and in 16 American Heart Association (AHA) myocardial segments. In phantom, Multitasking-SMS T1/T2 measurements showed substantial correlation (*R*^2^ > 0.996) and excellent agreement [intraclass correlation coefficients (ICC) ≥ 0.999)] with reference measurements. In healthy volunteers, Multitasking-SMS T1/T2 maps reported similar myocardial T1/T2 values (1,215 ± 91.0/41.5 ± 6.3 ms) to the reference myocardial T1/T2 values (1,239 ± 67.5/42.7 ± 4.1 ms), with *P* = 0.347 and *P* = 0.296, respectively. Bland–Altman analyses also demonstrated good *in vivo* repeatability in both the multitasking and references, with segment-wise coefficients of variation of 4.7% (multitasking T1), 8.9% (multitasking T2), 2.4% [modified look-locker inversion recovery (MOLLI)], and 4.6% (T2-prep FLASH), respectively. In summary, multitasking-SMS is feasible for free-breathing, non-ECG, myocardial T1/T2 quantification in 16 AHA segments over 3 short-axis slices in 3 min. The method shows the great potential for reducing exam time for quantitative CMR without ECG or breath-holds.

## Introduction

Cardiac magnetic resonance (CMR) imaging is rapidly evolving toward quantitative multiparameter measurement for myocardial tissue characterization (1–6). Quantitative myocardial T1 and T2 mapping techniques are especially useful for tissue characterization, clinical diagnosis, and disease monitoring (7–19). For example, T1 is sensitive to amyloidosis (11–13), fibrosis (15, 17), and inflammation (20); T2 is sensitive to water content in tissue, characterizing myocardial edema (16, 21), ischemia (21), inflammation (14), sarcoidosis (22), and more. Quantitative imaging techniques also enable comparison between patients scanned with differing scanners or timepoints and are therefore promising imaging biomarkers for multi-center or longitudinal studies (23).

Conventional cardiac T1 (24–27) and T2 (28) mapping techniques are inherently inefficient because (1) they rely on breath-holds (often one per slice, with pauses between acquisitions for patients to recover before the next breath-hold) and pauses in acquisition (*via* ECG triggering) to avoid respiratory and cardiac motion; and (2) are performed in series (slice-by-slice, biomarker-by-biomarker) instead of simultaneously. This approach becomes impractical for patients having difficulty holding their breath or for whom ECG triggering fails. Respiratory gating (29) is an alternative to breath-holding, but typically comes with low scan efficiency as well.

Multidimensional continuous-acquisition methods, such as MR Multitasking (5), have shown promise for free-breathing, non-ECG, simultaneous parameter mapping by simultaneously resolving the overlapping dynamics (i.e., cardiac/respiratory motions, relaxations, etc.) involved in quantitative CMR. Multitasking uses a low-rank tensor (LRT) imaging approach with subspace modeling to address the curse of dimensionality associated with imaging multiple motions and relaxations. This approach removes the conventional inefficiencies of scan pauses and serial biomarker acquisition; however, 2D multitasking still uses serial slice acquisition, and so has the same slice coverage inefficiencies as conventional scans. Clinical protocols for quantitative CMR typically include T1 and T2 maps in mid, basal, and apical short-axis slices. Therefore, slice-by-slice 2D multitasking is not fully efficient for the simultaneous acquisition of all biomarkers at all slices. Volumetric 3D Multitasking (30, 31) has been preliminarily demonstrated over 14 short-axis slices (whole ventricle coverage) with 1.4 mm ×1.4 mm ×8 mm resolution in 9:14 min, but provides more slice coverage than is currently used in clinical protocols, at the expense of scan time.

Simultaneous multislice (SMS) imaging (32, 33) has the potential to address the slice inefficiencies of 2D Multitasking without the scan time extension required by full 3D coverage. Here, we redesign MR Multitasking sampling and reconstruction to incorporate SMS imaging, performing three-slice myocardial T1/T2 mapping in a 3 min, non-ECG, free-breathing MRI scan. The repeatability of quantitative measurements and the agreement with reference approaches were evaluated in phantom and in healthy volunteers.

## Materials and Methods

### Cardiac MR Multitasking Framework

#### Pulse Sequence Design With SMS Acceleration

**Figure 1:**
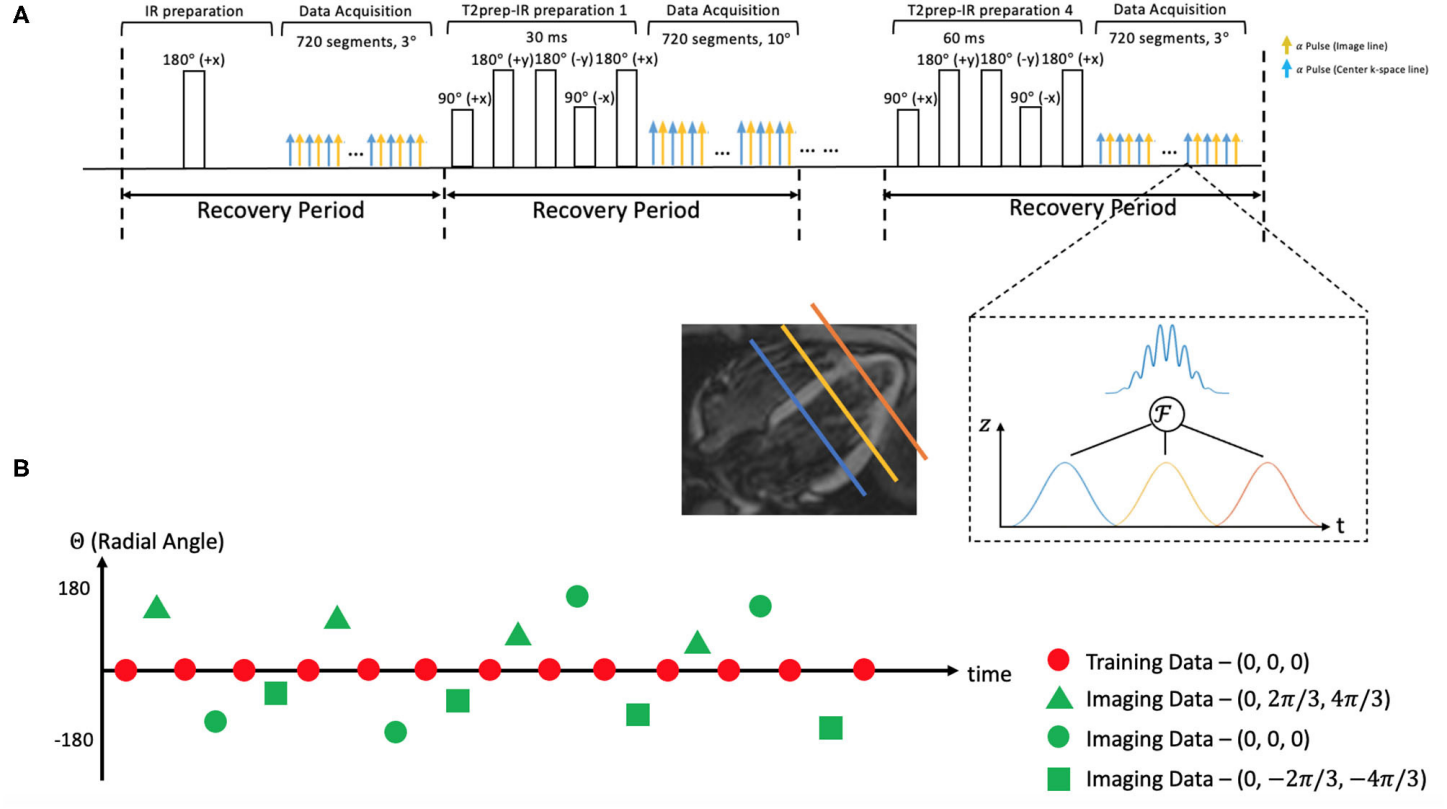
**(A)** Schematic diagram of the proposed 2D magnetic resonance (MR) Multitasking sequence with simultaneous multislice (SMS)-acceleration, where 5 different preparations (IR and T2prep-IR with 4 different preparation times) are repeated throughout the scan. Each FLASH excitation pulse can excite three slices simultaneously. **(B)** k-Space sampling demonstration. Imaging data are collected with a 2D radial trajectory, and they are incremented by a golden angle (i.e., 111.24°) for each readout. Training data periodically sample the center k-space line every other readouts. Three short-axis slices are excited simultaneously with different phase modulation schemes, resulting in a 2π/3, 0, –2π/3 shift in their phase increment, respectively.

A prototype MR pulse sequence was developed based on our previous CMR Multitasking implementations (5). T2prep-IR pulses were employed to generate the T1 and T2 contrasts (as shown in Figure 1). The T2 prep-IR module was modified from an adiabatic T2-preparation module (34) by adding one adiabatic 180 inversion pulse after the 90 tip-up pulse in the T2-preparation module to achieve the inversion effect. The sequence cycled through five T2 prep-IR durations and the special case of “0 ms” preparation duration used only the IR pulse without any T2 preparation. A continuous-acquisition FLASH sequence collected readouts throughout the entire T1 recovery process. Successive recovery periods alternated between two FLASH excitation flip angles to allow B1^+^–and through-plane-motion–robust T1 mapping (35). Interleaving five T2prep-IR durations while also interleaving two flip angles produces a cycle of 10 T2prep-IR duration/flip angle combinations, which was repeated throughout the scan. The sequence employed radial k-space sampling, alternating between imaging data readouts incremented by the golden angle (111.24°) and between training data at a fixed radial angle (0°). The image data target spatially resolvable information through (0° and 360°) angular coverage of k-space, whereas the training data collect one projection line at a high temporal sampling rate to facilitate self-gating and will be used to define temporally resolved model parameters during image reconstruction.

A multiband factor of three was used to acquire three slices at the same time. The simultaneous excitation of multiple slices was achieved by superimposing single-band excitation pulses at equally spaced center frequencies, corresponding to equally spaced slice locations. The phase of each band was cycled by different increments (−2π/3, 0, and +2π/3), mimicking the discrete Fourier transform and defining a discrete *k*_*z*_ dimension. This encoding scheme is a generalization of the controlled aliasing in volumetric parallel imaging (CAIPIRINHA) technique (36). SMS encoding was applied on every FLASH excitation pulse to always excite three slices simultaneously. No phase cycling was used on the mid-ventricular slice, the +2π/3 phase increment was used on the basal slice, and the −2π/3 phase increment on the apical slice. The phase cycle was incremented by one step for each imaging data readout, corresponding to linear *k*_*z*_ encoding; no phase modulation was used for the training data, corresponding to *k*_*z*_ = 0. The training data contain contributions from all 3 slices with matched phases, akin to a projection along the slice direction.

#### Low-Rank Tensor Imaging Model

The images acquired in the Multitasking framework can be represented as a 5-way tensor A (5, 37). Multitasking conceptualizes different sources of image dynamics involved in quantitative cardiovascular imaging as an image array/tensor with images sorted according to different time dimensions. These image dynamics (e.g., cardiac, respiratory motions, T1/T2 relaxations) overlap in real-time, but by organizing them into a tensor and exploiting the correlation between images, Multitasking can simultaneously resolve all of them. As a result, we can capture and view different image dynamics along different time dimensions.

**Figure 2:**
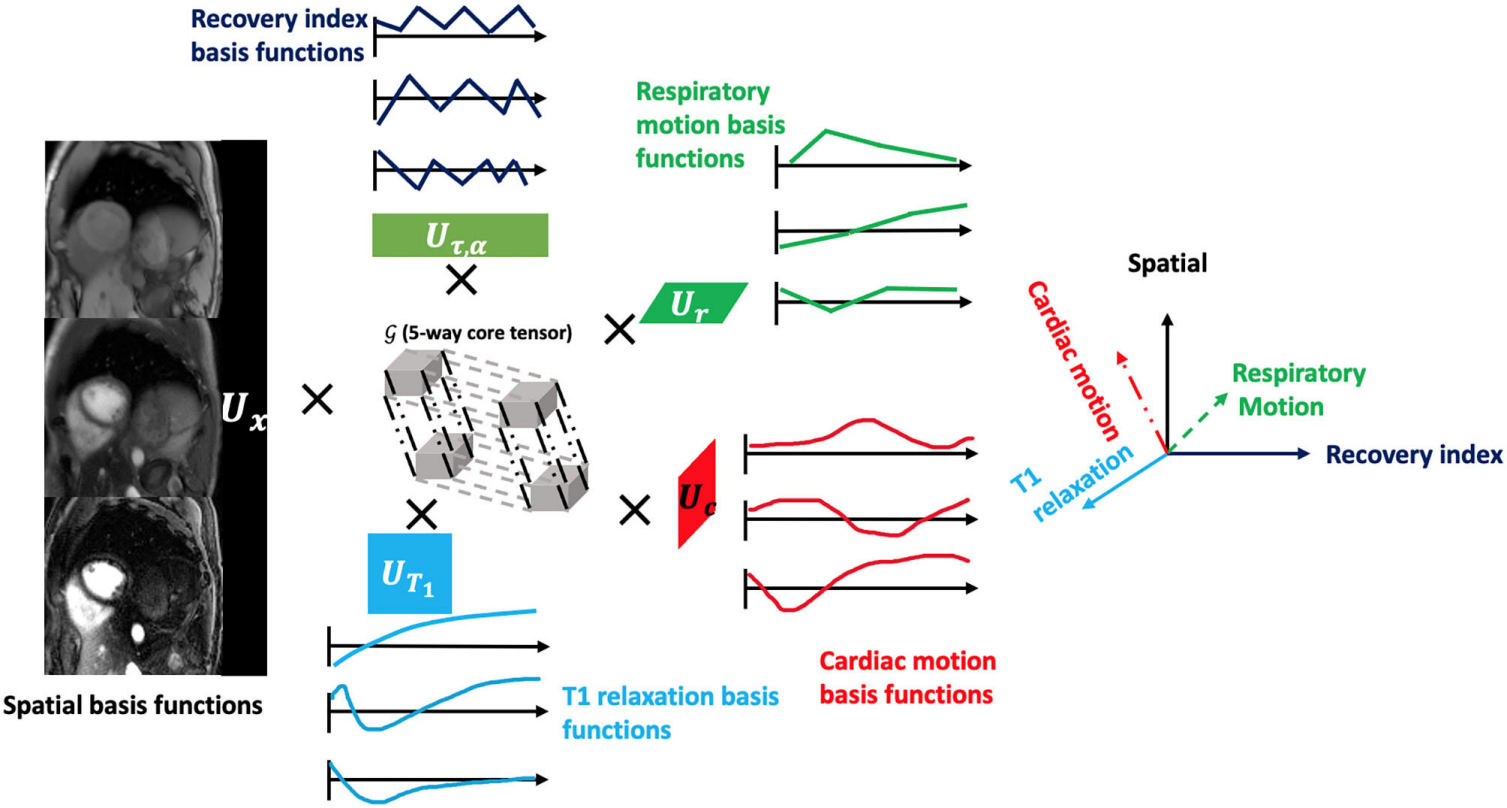
The framework of the low-rank tensor (LRT) imaging model. The underlying image can be represented as a 5-way tensor with one spatial dimension and 4 time dimensions representing 4 dynamic processes: T1 relaxation process, recovery weights with different T2prep-IR duration and flip angles, respiratory motion, and cardiac motion. With the LRT image model, the tensor can be factorized into five factor matrices with much smaller sizes, reducing the degrees of freedom for the LRT recovery problem.

We model A as a LRT, leveraging image correlation laterally along each of the *N* time dimensions and diagonally throughout the multidimensional temporal space, reducing the images to the product of a small core tensor and five factor matrices:


(1) $$\begin{array}{r} A=G\times_{1}{\text{U}}_{\text{x}}\times_{2}{\text{U}}_{T_{1}}\times_{3}{\text{U}}_{\tau ,\alpha }\times_{4}{\text{U}}_{c}\times_{5}{\text{U}}_{r}, \end{array}$$

where **U**_**x**_ contains spatial basis functions with voxel location index **r** = (*x, y, z*), **U**_*T*_1__ contains basis functions for the T1 relaxation, **U**_τ, α_ contains basis functions that index the 10 different recovery modules with varying T2prep-IR duration τ and flip angle α combinations, **U**_*c*_ contains cardiac motion basis functions, **U**_*r*_ contains respiratory motion basis functions, and G is the core tensor governing the interaction between factor matrices. This constrains the image tensor A to the intersection of the five low-dimensional subspaces spanned by the **U** matrices. The factor matrices and core tensor have far fewer elements than the full image tensor A, which reduces the degrees of freedom for the LRT recovery problem and allows memory-efficient image reconstruction. A diagram of the LRT imaging model is shown in Figure 2.

#### Image Reconstruction

Image reconstruction in the CMR Multitasking framework is divided into the following steps: (1) preliminary “real-time” (ungated) image reconstruction; (2) predetermining the temporal basis functions in **U**_*T*_1__ and **U**_τ, α_ from a training dictionary of signal curves; (3) cardiac and respiratory binning of the real-time images; (4) determining the motion bases and core tensor from the training data; and finally, (5) solving for the spatial coefficients **U**_**x**_ from the imaging data.

#### Real-Time Image Reconstruction

“Real-time” (i.e., one single time dimension representing elapsed time) image reconstruction generates ungated images with a low-rank matrix imaging strategy (37), to facilitate image-based binning. The temporal basis functions are estimated from the singular value decomposition (SVD) of the training data, and the spatial coefficients are estimated by least-squares fitting to the imaging data (37).

#### Dictionary Generation for T1 and Recovery Index Basis Functions

We generated a training dictionary of feasible T2-IR-FLASH signal curves governed by the Bloch equations, with a range of variable T1/T2 values, B1 inhomogeneities, and inversion efficiencies (30, 31). We used 21 T1 values logarithmically spaced between 100 and 3,000 ms, 21 T2 values logarithmically spaced between 10 and 3,000 ms, seven B1+ efficiency values between 0 and 1.5 modulating the excitation flip angles, and seven inversion efficiency factors controlling the effects of inversion efficiency for the IR and T2prep-IR pulses. The T1 and recovery index relaxation basis functions in **U**_*T*_1__ and **U**_τ, α_ are estimated from the SVD of this training dictionary.

#### Respiratory and Cardiac Motion Binning

The respiratory and cardiac motion binning algorithm is derived from the methods described in the original MR Multitasking work (5). Briefly, we used an unsupervised machine learning approach to identify motion states by employing a modified k-means clustering algorithm incorporating a low-rank NMR relaxation model (i.e., the known **U**_*T*_1__ and **U**_τ, α_) to address the variable contrast weighting of the training data. We used 6 respiratory bins and 20 cardiac bins in the binning procedure.

#### Temporal Factor Estimation

Once the motion states have been identified, the training data can be reorganized as a 5-way tensor $D_{tr}$ which shares temporal factors and core tensor with the image tensor A. These training data will cover several—but not all—combinations of cardiac phase, respiratory phase, recovery index, and inversion time. To recover missing combinations, we apply an LRT completion algorithm, solving the optimization problem below:


(2) $$\begin{array}{l} {\hat{D}}_{tr}=\;\min\;{\left\|{\text{d}}_{tr}-\Omega_{tr}\left(D_{tr}\right)\right\|}_{2}^{2}w\\ \;D_{tr},\\ \;D_{tr,\left(2\right)}\in range\left(U_{T_{1}}\right)\\ \;D_{tr,\left(3\right)}\in range\left(U_{\tau ,\alpha }\right)\\ \;+\lambda \sum i=1,4,5{\left\|D_{tr,(i)}\right\|}_{*}+R_{t}(D_{tr}), \end{array}$$

where **d**_*tr*_ is the collected training data, Ω_*tr*_(·) is the sampling operator for the training dataset, **D**_*tr*, (*i*)_ is the mode-*i* unfolding of the training tensor, ||·||_*_ denotes the matrix nuclear norm, and *R*_*t*_(·) is a temporal regularizer, which was chosen as temporal total variation (TV) along the respiratory and cardiac dimensions in this work (38). $R_{t}\left(D_{tr}\right)$ in Eq. (2) can be expressed as


(3) $$\begin{array}{r} R_{t}\left(D_{tr}\right)=\lambda_{c}||{\text{D}}_{tr,\left(4\right)}|{|}_{1}+\lambda_{r}||{\text{D}}_{tr,\left(5\right)}|{|}_{1}, \end{array}$$

where λ_*c*_ and λ_*r*_ are the two regularization parameters that control the TV smoothing along the cardiac and respiratory dimensions.

Once the training data tensor ${\hat{D}}_{tr}$ is complete, the core tensor G and the remaining unknown temporal factor matrices **U**_*c*_ and **U**_*r*_ are extracted from the higher-order SVD (HOSVD) (39) of $D_{tr}$. At this stage, the core tensor and all temporal factor matrices are known, permitting the definition of a combined temporal factor tensor $\Phi =G\times_{2}{\text{U}}_{T_{1}}\times_{3}{\text{U}}_{\tau ,\alpha }\times_{4}{\text{U}}_{c}\;\times_{5}{\text{U}}_{r}.$

#### Spatial Factor Estimation

The spatial factor **U**_**x**_ was then recovered by fitting the known Φ to the acquired imaging data **d**, using the following optimization problem:


(4) $$\begin{array}{r} {\hat{\text{U}}}_{\text{x}}=\arg\underset{{\text{U}}_{\text{x}}}{\min}||\text{d}-\Omega \left(\Phi \times_{1}\text{FS}{\text{U}}_{\text{x}}\right)|{|}_{2}^{2}+\text{R}\left({\text{U}}_{\text{x}}\right), \end{array}$$

where Ω is the undersampling operator, **F** is the Fourier transform operator comprising non-uniform in-plane Fourier encoding and Fourier slice encoding, **S** is the coil sensitivity operator, and *R*(·) is an optional regularization functional to promote transform sparsity (chosen as a wavelet transform in this implementation). *R*(**U**_*x*_) in Eq. (4) can be expressed as


(5) $$\begin{array}{r} R\left({\text{U}}_{x}\right)=\lambda_{w}{\left||{\text{W}\text{U}}_{x}|\right|}_{1}, \end{array}$$

where **W** is the wavelet operator and λ_*w*_ is the regularization parameter that controls the wavelet sparsity. Once Φ and **U**_**x**_ have both been determined, the final reconstructed image can be calculated as $A=\Phi \;\times_{1}{\text{U}}_{\text{x}}$.

#### Multiparametric Mapping

The signal equation at the *k*^*th*^ recovery period of the Multitasking-SMS pulse sequence is:


(6) $$\begin{array}{r} \;s\left(A,B,T_{1},\;T_{2},\beta \right)=A\frac{1-e^{-TR/T_{1}\;}}{1-e^{-TR/T_{1}}\cos\left(\beta \alpha_{k}\right)}\\ \cdot \left[1+\left(B{Q_{k}e}^{-\frac{\tau }{T_{2}}}-1\right){\left(e^{-\frac{TR}{T_{1}}}\cos\left(\beta \alpha_{k}\right)\right)}^{n}\right]\cdot \sin\left(\beta \alpha_{k}\right), \end{array}$$

with amplitude factor *A*, IR/T2prep-IR pulse efficiency *B*, FLASH readout interval *TR*, flip angle for the *k*th recovery period α_*k*_, B1+ field weights β (to account for B1+ inhomogeneity), and recovery time points *n* = 1, 2, …, *N* (where *N* is the total number of excitations in each recovery period). The *Q*_*k*_ absorbs the effects of having inverted the magnetization from the steady-state for the previous recovery period's excitation flip angle. Assuming a steady-state established at the final readout of each recovery period, *Q*_*k*_ is expressed as


(7) $$\begin{array}{r} Q_{k}=\frac{1-e^{-TR/T_{1}\;}\cos\left(\beta \alpha_{k}\right)}{1-e^{-TR/T_{1}}\cos\left(\beta \alpha_{k-1}\right)}. \end{array}$$

The native T1 and T2 measurements can be estimated from the signal model in Eqs. (6) and (7). Our previous work (35) showed the value of a dual flip-angle signal model for B1+ robust T1 mapping.

### Phantom Study

An International Society for Magnetic Resonance in Medicine/National Institute of Standards and Technology (ISMRM/NIST) phantom (40) (model 130, High Precision Devices, Boulder, Colorado) was imaged on a 3T scanner (MAGNETOM Vida, Siemens). The layer with the vials closest to the T1 and T2 values for myocardium (T1 ∈ [200, 2,500] ms; T2 ∈ [20, 800] ms) was used in the study.

The proposed 2D Multitasking-SMS sequence was applied, as well as four reference methods: modified look-locker inversion recovery (MOLLI) 5(3)3 (41), T2-prepared fast low angle shot (T2-prep FLASH) mapping method (common product sequences used in the heart), and the gold standard static T1 and T2 mapping sequences inversion recovery spin echo (IRSE-T1) for T1 mapping, and T2-weighted spin-echo (SE-T2) for T2 mapping.

The following scan parameters were used for the proposed 2D Multitasking-SMS sequence: Field of View (FOV) = 270 mm ×270 mm (with 2-fold readout oversampling, the acquired FOV = 540 mm ×540 mm); spatial resolution = 1.7 mm ×1.7 mm ×8 mm; 3 slices with a multiband factor of 3; TR/TE = 3.5/1.6 ms; flip angle = 3 and 10; T2 preparation times = 0, 30, 40, 50, and 60 ms (with 0 corresponding to a standard IR pulse); recovery period = 2.5 s; scan time = 3 min 3 s. The 2D MOLLI imaging parameters were: Repetition Time/Echo Time (TR/TE) = 2.7/1.1 ms; flip angle = 35; FOV = 220 mm ×220 mm; in-plane resolution = 1.4 mm ×1.4 mm; slice thickness = 8 mm. The 2D T2-prep FLASH imaging parameters were: TR/TE = 3.3/1.4 ms; flip angle = 12; FOV = 220 mm ×220 mm; in-plane resolution = 1.4 mm^2^ ×1.4 mm; slice thickness = 8 mm; T2 preparation times = 0, 35, and 55 ms. The IR-SE T1 protocol parameters were: FOV = 280 mm ×192 mm; in-plane resolution = 1.4 mm ×1.4 mm; slice thickness = 5 mm; TI = 150, 300, 500, 800, 1,200, 1,600, 2,000, and 4,500 ms. The SE-T2 protocol parameters were: FOV = 280 mm ×192 mm; in-plane resolution = 1.4 mm ×1.4 mm; slice thickness = 5 mm; TE = 15, 25, 45, 70, 100, 140, 180, 250, and 350 ms.

Linear regression, the Bland–Altman analyses, and intraclass correlation coefficients (ICC) with a two-way mixed model were performed on the vials with relevant T1 and T2 values (T1 <2,000 ms; T2 <120 ms) to evaluate the quantitative agreement between Multitasking and reference measurements. Pairwise *t*-tests were also performed to evaluate measurement biases, with a significance level of 0.05.

### *In-vivo* Study

Healthy volunteer studies were approved by the institutional review board of Cedars-Sinai Medical Center. All subjects gave written informed consent before MRI. *N* = 10 human volunteers (3 men and 7 women, age 36.7 ± 12.3) were imaged on a 3T scanner (MAGNETOM Vida, Siemens) with an 18-channel body coil.

The 2D Multitasking-SMS pulse sequence imaged three short-axis slices over the left-ventricle, base, mid, and apex. It was applied twice to test scan-rescan repeatability. The scan parameters were the same as used in the phantom study. A 2-step fitting procedure was used to determine parameter maps. Step 1 estimates β and T2 from Eq. (6), and Step 2 uses the known β to fit T1 from the 3° recovery curve only, for which the Look–Locker effect is reduced.

The 2D single-slice multitasking (i.e., multitasking-SS) pulse sequence was also applied to sequentially image the same three short-axis slices. The scan parameters were: FOV = 270 ×270 mm (with two-fold readout oversampling, the acquired FOV = 540 mm ×540 mm); spatial resolution = 1.7 mm ×1.7 mm ×8 mm; TR/TE = 3.5 ms/1.6 ms; flip angle = 5; T2 preparation times = 0, 30, 40, 50, and 60 ms; recovery period = 2.5 s; scan time per slice = 1 min 31 s (4 min 33 s for 3 slices).

Reference 2D T1 maps with MOLLI and 2D T2 maps with T2-prep FLASH (1.4 mm ×1.4 mm ×8.0 mm) were acquired at both systole and diastole during end-expiration breath-holds and were also collected twice. The scan parameters of the reference sequences were the same as used in the phantom study.

T1 and T2 maps were segmented at the end-expiration respiratory and end-diastolic cardiac phases, using the AHA 16-segment model by drawing epi- and endocardial contours in commercially available software (CVI42; Circle Cardiovascular Imaging, Calgary, Alberta, Canada) (42).

Measurement of global and segmental myocardial T1/T2 in all healthy volunteers were compared between the Multitasking-SMS approach and the reference approaches. The pairwise *t*-tests were performed with a significance level of 0.05. Repeatability was evaluated using Bland–Altman analyses and coefficients of variation (CoVs) between the first and second scans of the proposed method and of reference methods. Global and segment-wise CoVs were calculated to assess repeatability. Global CoVs were calculated as the standard deviation of average Left Ventricle (LV) myocardial T1/T2 between two scans, divided by the mean T1/T2 for each subject, and were root-mean-square (RMS)-aggregated over all 10 subjects to provide an overall summary of global repeatability. Segment-wise CoVs were calculated as the standard deviation between two scans for each LV myocardial segment, and were first RMS-aggregated over segments to calculate the segment-wise CoV and divided by the mean T1/T2 for each individual subject; segment-wise CoVs between methods, with a significance level of 0.05. Segment-wise CoVs were then RMS-aggregated over all 10 subjects to provide an overall summary of segment-wise repeatability.

To determine the impact of dual-flip-angle SMS imaging on the accuracy and precision of multitasking measurements, pairwise *t*-tests were used to compare the segmental T1/T2 values, segment-wise Signal-to-noise ratio (SNR), and segment-wise SNR efficiency between Multitasking-SS and Multitasking-SMS. The segment-wise SNR was calculated as the mean T1/T2 within each segment divided by the voxelwise standard deviation of T1/T2 within that segment. SNR values were transformed into 3-slice SNR efficiency values by dividing by the square root of the total scan time required to collect 3 slices (4.5 min for Multitasking-SS and 3 min for Multitasking-SMS).

### Materials and Software

All Multitasking image reconstructions were performed on a Linux workstation with a 2.90 GHz Intel Xeon processor in MATLAB 2018a (MathWorks, Natick, Massachusetts). Statistical analyses were performed using IBM SPSS Statistics (Armonk, New York, USA).

## Results

### Phantom Results

**Figure 3:**
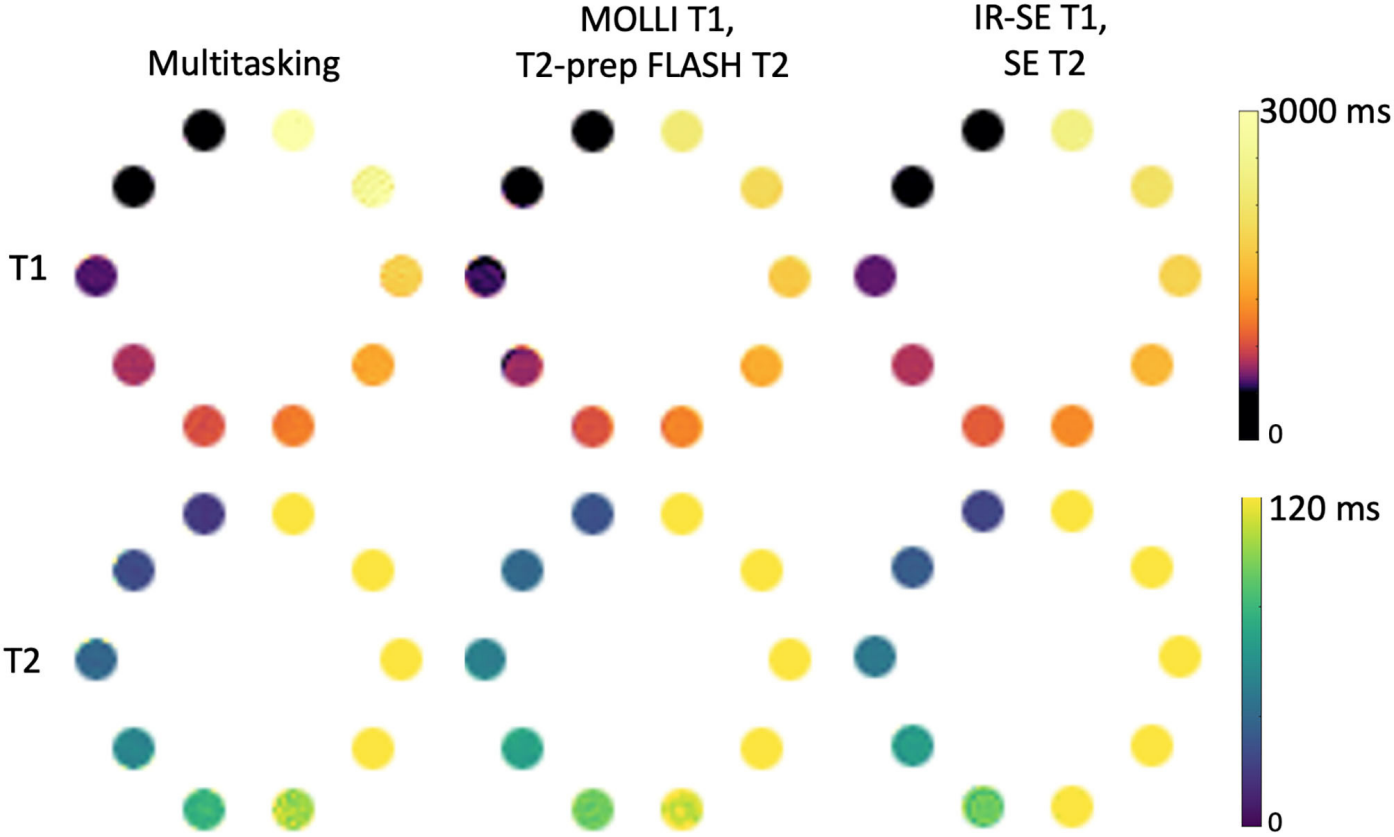
T1 and T2 measurements of the ISMRM/NIST phantom using the Multitasking-SMS method and the reference methods [modified look-locker inversion recovery (MOLLI), T2-prep FLASH methods; IRSE-T1 and SE-T2 methods]. 8 vials with T1 < 2,000 ms are used for T1 analysis, and 5 vials with T2 < 120ms are used for T2 analysis.

Phantom T1 and T2 maps obtained from the 2D MOLLI, T2-prep FLASH, IRSE-T1, SE-T2, and the Multitasking-SMS approaches are shown in Figure 3. Table 1 summarizes the ICC and Paired *t*-test results between Multitasking-SMS/MOLLI/T2-prep FLASH and gold standard IRSE/SE measurements. Multitasking-SMS measurements and IRSE/SE measurements showed excellent agreement with ICC = 0.999 for both T1 and T2. All pairwise method comparisons showed statistically significant biases.

**Table 1:** Intraclass correlation coefficient (ICC) and *P*-values of the paired *t*-test for comparison analysis between cardiac mapping methods and the gold standard and reference values across vials in the ISMRM/NIST phantom.

	**ICC**	***P*-value (Paired *T*-Test)**
MOLLI T1 vs. IRSE T1	0.999	<0.001
Multitasking T1 vs. IRSE T1	0.999	0.011
T2-prep FLASH T2 vs. SE T2	0.999	0.010
Multitasking T2 vs. SE T2	0.999	0.006

**Figure 4:**
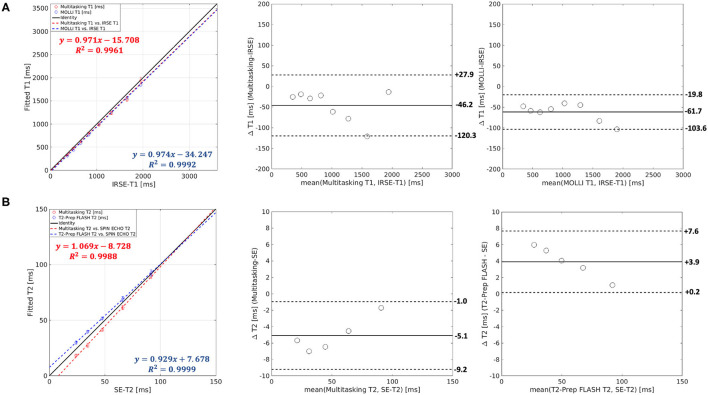
**(A)** Both Multitasking-SMS and MOLLI showed high *R*^2^ values against inversion recovery spin echo (IRSE)-T1 in the linear regression. The T1 bias and limits of agreement were −46.2 ± 74.1ms for Multitasking-SMS and IRSE-T1, and −61.7 ± 41.9 ms for MOLLI and IRSE-T1. **(B)** Both Multitasking-SMS and T2-prep FLASH showed a high R^2^ value against SE-T2. The T2 bias and limits of agreement were −5.1 ± 4.1 ms for the Multitasking-SMS and SE-T2, and 3.9 ± 3.7 ms for T2-prep FLASH and SE-T2.

Scatter plots and Bland–Altman plots of the T1 and T2 values in the relevant vials are shown in Figure 4. T1 measurements from Multitasking-SMS and MOLLI were each highly correlated (*R*^2^ > 0.996) with the reference 2D IRSE-T1 acquisition. The 95% limits of agreement of the T1 values were 46.2 ± 74.1 ms for Multitasking-SMS and IRSE-T1, and −61.7 ± 41.9 ms for MOLLI and IRSE-T1. T2 measurements from Multitasking-SMS and T2-prep FLASH were also each highly correlated (*R*^2^ > 0.998) with the reference 2D SE-T2 acquisition. The 95% limits of agreement of the T2 measurements were −5.1 ± 4.1ms for Multitasking-SMS and SE-T2, and 3.9 ± 3.7ms for T2-prep FLASH and SE-T2.

### *In-vivo* Results

**Figure 5:**
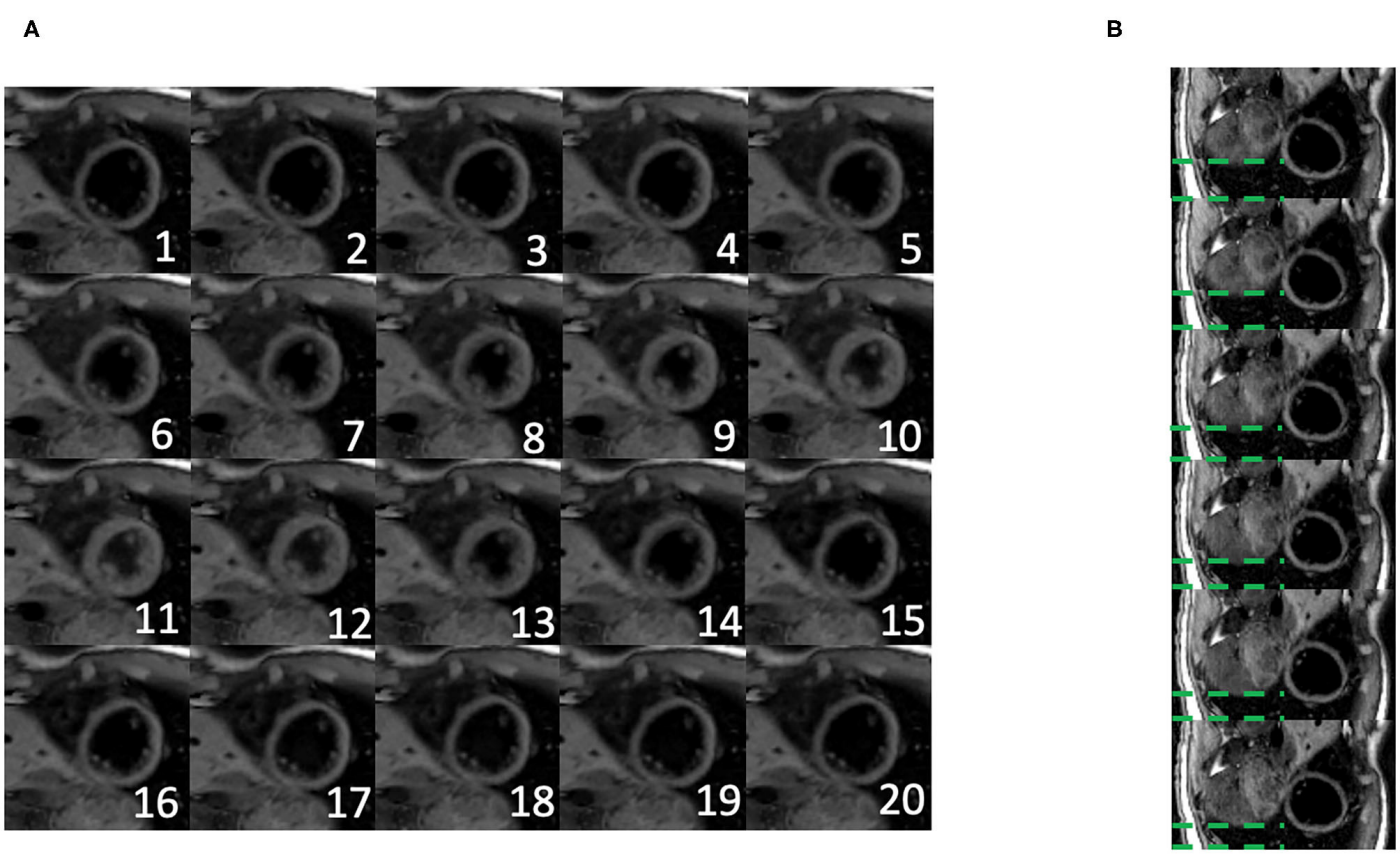
**(A)** Twenty cardiac phases are generated after the binning procedure. **(B)** Six respiratory phases are generated after the binning, the displayed images show the exhalation process. The green dash line represents the distance between the liver dome and the bottom of the image. The liver dome position approaches the bottom of the image during exhalation.

**Figure 6:**
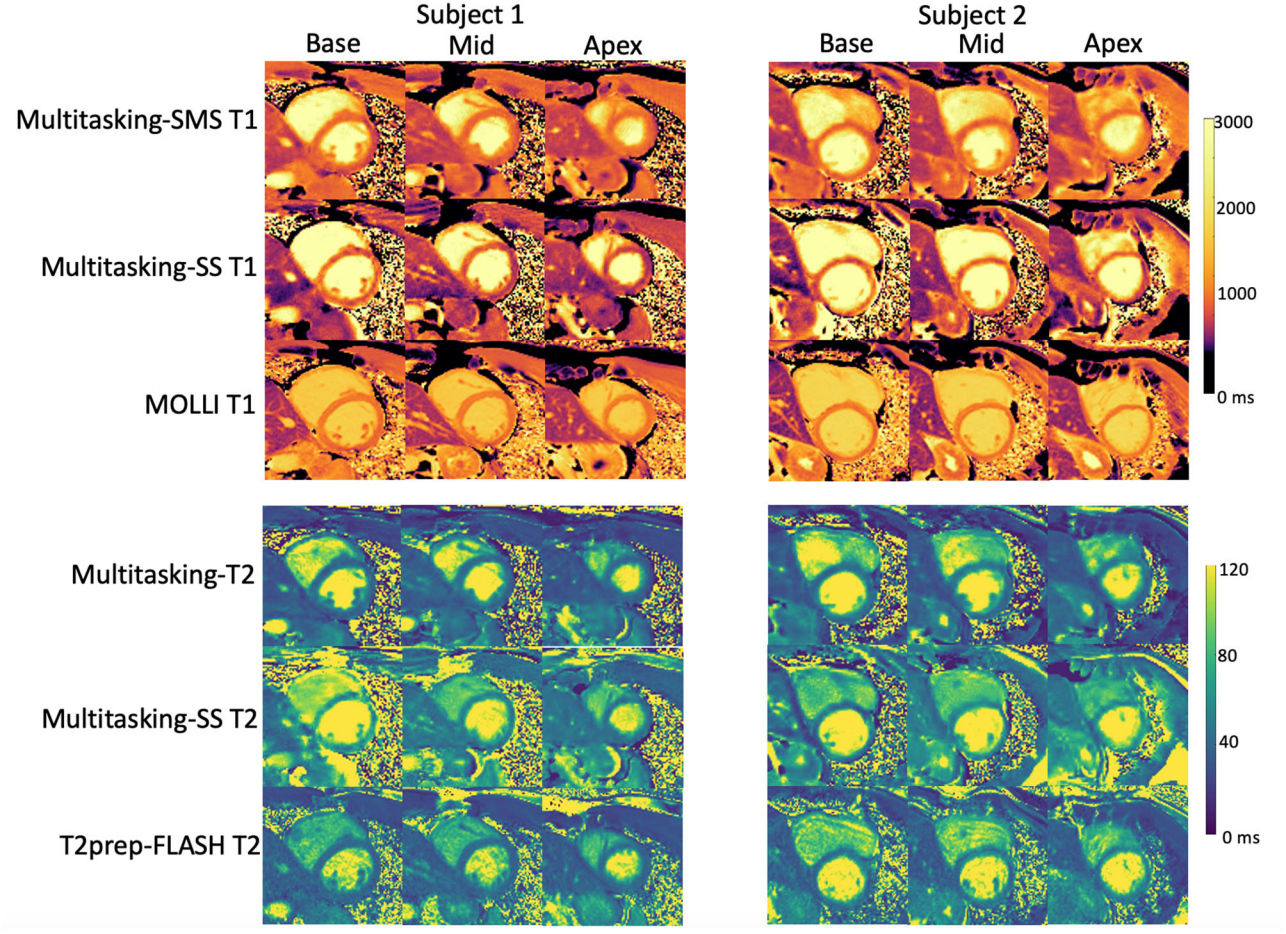
Comparison between T1/T2 maps obtained with the proposed 2D Multitasking-SMS, the original Multitasking-SS, and the standard MOLLI/T2-prep FLASH approaches in two healthy subjects. The acquired Multitasking T1/T2 maps were the same slice position as the reference maps acquired in the short axis.

**Figure 7:**
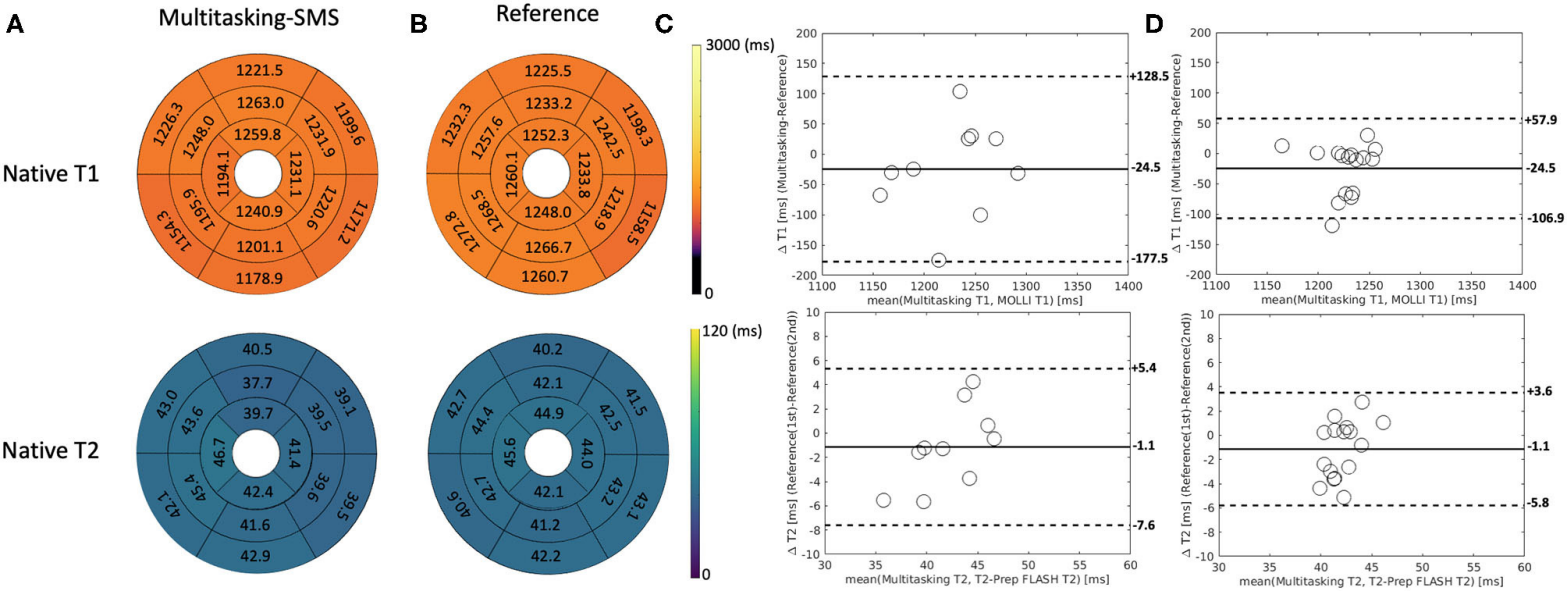
**(A,B)** The 16-segment AHA model for the proposed Multitasking-SMS T1/T2 maps and the reference T1/T2 maps in the myocardium in all 10 healthy subjects. **(C)** The Bland-Altman plot compares the subject-wise global myocardium T1/T2 differences in 10 healthy subjects. **(D)** The Bland-Altman plot compares the segment-wise T1/T2 differences in 10 healthy subjects. The dash lines represent 95% limits of agreement, and the solid lines represent mean bias.

Figure 5 shows the cardiac and respiratory phases detected in the Multitasking-SMS framework in one subject. Motion videos are provided in Supplementary Videos 1, 2. T1 and T2 mapping results from 2D Multitasking-SMS, 2D Multitasking-SS, and reference methods in two healthy subjects (including 3 short-axis slices) are shown in Figure 6. Additional cardiac phases from Multitasking-SMS are shown in Supplementary Figures 1, 2. Example fitted B1+ field maps (β) and inversion efficiency maps (*B*) obtained from Multitasking-SMS are given in Supplementary Figure 6. Figures 7A,B show the mean T1/T2 values in each of the 16 AHA segments across all 10 healthy subjects as a bull's eye plot, for the Multitasking-SMS and the reference methods. The Bland–Altman plots in Figures 7C,D further compare the Multitasking-SMS T1 values with the reference T1 values estimated by MOLLI, and the Multitasking-SMS T2 values with the reference T2 values estimated by T2-prep FLASH. Both subject-wise (averaged from whole myocardium for each subject, 10 values) and segment-wise (averaged from all subjects for each segment, 16 values) T1/T2 measurements are compared between Multitasking-SMS and references. Supplementary Figure 3 shows these plots for all subject/segment combinations. Multitasking-SMS measured similar global T1 (1215 ± 91.0 ms) and T2 (41.5 ± 6.3 ms) values to MOLLI (1239 ± 67.5 ms) and T2-prep FLASH (42.7 ± 4.1 ms), with *P* = 0.347 and *P* = 0.296, respectively.

**Figure 8:**
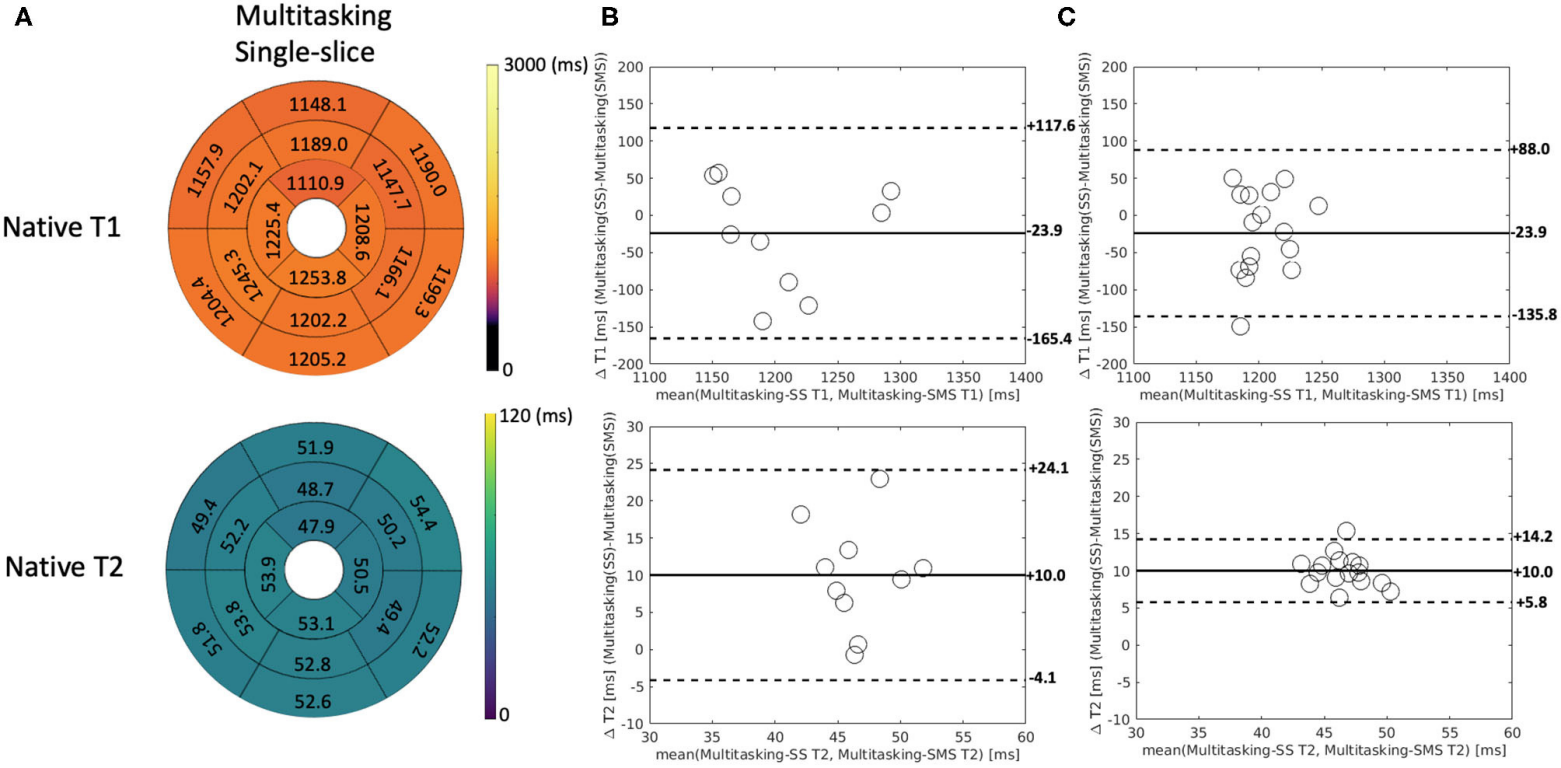
**(A)** The 16-segment AHA model for the 2D single-slice Multitasking T1/T2 maps in the myocardium in all 10 healthy subjects. **(B)** The Bland-Altman plot compares the subject-wise global myocardium T1/T2 differences between Multitasking-SS and Multitasking-SMS in 10 healthy subjects. **(C)** The Bland-Altman plot compares the segment-wise T1/T2 differences between Multitasking-SS and Multitasking-SMS in 10 healthy subjects. The dash lines represent 95% limits of agreement, and the solid lines represent mean bias.

Additionally, Multitasking-SS T1/T2 mapping results were compared to the Multitasking-SMS measurements in Figure 8. Supplementary Figure 4 further shows the Bland-Altman comparisons for all subject/segment combinations. 2D single-slice Multitasking measured similar global T1 (1,191 ± 106.5 ms; *P* = 0.323) and higher T2 (51.6 ± 7.2 ms; *P* = 0.002) values compared to Multitasking-SMS T1/T2. The significant bias in T2 also exists between Multitasking-SS T2 and reference T2-prep FLASH measurements (*P* <0.001).

**Figure 9:**
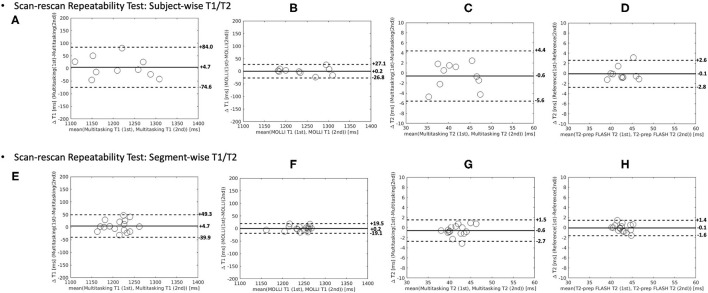
The Bland–Altman plots comparing measurements from 1st and 2nd Multitasking-SMS scans and reference scans in subject-wise T1/T2 **(A–D)** and segment-wise T1/T2 **(E–H)**. Multitasking-SMS T1/T2 repeatability analysis are shown in **(A,C,E,G)**. Reference T1/T2 repeatability analysis are shown in **(B,D,F,H)**. The dash lines indicate the 95% limits of agreement and the solid line indicates mean bias.

The Bland–Altman plots in Figure 9 show the subject-wise and segment-wise scan-rescan repeatability of multitasking-SMS and reference T1/T2 measurements. Supplementary Figure 5 shows the Bland–Altman plots for all the subject/segment combinations. The RMS global CoVs of subject-wise T1/T2 values were 2.3% (multitasking T1), 4.4% (multitasking T2), 0.7% (MOLLI), 2.1% (T2-prep FLASH), respectively. The RMS segment-wise CoVs across all 16 segments' T1/T2 values in the 10 subjects were 4.7% (multitasking T1), 8.9% (multitasking T2), 2.4% (MOLLI), and 4.6% (T2-prep FLASH). Segment-wise CoVs were significantly larger for multitasking T1 than MOLLI T1 (*P* = 0.002), and significantly larger for multitasking T2 than T2-prep FLASH (*P* = 0.001).

The average SNR of multitasking measurements were 11.9 (multitasking-SMS T1), 6.2 (multitasking-SMS T2), 6.0 (multitasking-SS T1), and 6.1 (multitasking-SS T2). The average 3-slice SNR efficiencies were 6.9 min^−1/2^ (multitasking-SMS T1), 3.6 min^−1/2^ (multitasking-SMS T2), 2.8 min^−1/2^ (multitasking-SS T1), and 2.9 min^−1/2^ (multitasking-SS T2). The pairwise *t*-tests showed that multitasking-SMS had significantly higher T1 SNR and T1/T2 SNR efficiency than multitasking-SS (*P* <0.001) and similar T2 SNR (*P* = 0.692).

## Discussion

In this study, a 2D SMS-accelerated, free-breathing, non-ECG, motion-resolved cardiac imaging method (i.e., multitasking-SMS) was introduced for simultaneous 2D myocardial T1/T2 mapping over three short-axis slices in 3 min. It represents several new developments that have not previously been a part of T1-T2 multitasking: (1) this is the first SMS acceleration with multitasking framework; and (2) the first use of a dual flip angle scheme interleaved with T2prep-IR blocks for B1+-robust T1-T2 mapping.

In the phantom study, the multitasking-SMS T1/T2 measurements and the typical cardiac mapping sequences MOLLI and T2-prep FLASH all showed statistically significant biases against the gold standard IRSE-T1 and SE-T2. For T1, multitasking-SMS and MOLLI both had small negative biases; for T2, multitasking-SMS and T2prep-FLASH had small biases in opposite directions (underestimation by multitasking-SMS and overestimation by T2prep-FLASH). All the comparisons showed ICC ≥ 0.999, reflecting high agreement with gold standard references.

In healthy volunteers, multitasking-SMS T1/T2 measurements reported similar myocardial T1/T2 values compared to the reference T1/T2 measurements in healthy volunteers. The T1/T2 estimations from all methods were in the normal range of many 3T MRI studies (43–45). Multitasking-SMS was less repeatable than MOLLI and T2-prep FLASH in healthy volunteers, but may be an attractive choice for mapping in subjects who cannot comply with breath-holds or for whom ECG triggering fails, or when co-registration between T1 and T2 maps is desired. Multitasking-SMS underestimated T2 and T2-prep FLASH overestimated T2 in the phantom, but they achieved similar T2 quantification *in vivo*. This may be related to the difference in T2-prep modules between multitasking-SMS and T2prep-FLASH (T2prep-IR vs. T2prep, respectively). These different modules may have different responses to motion, inhomogeneity, and flow that are present in the *in vivo* scans, which could change their behavior relative to the phantom scans.

2D multitasking-SS scans from our original work (5) were also applied sequentially on the same short-axis slice locations. Multitasking-SS T2 values were significantly shorter than both the Multitasking-SMS and the T2prep-FLASH T2 values—which were not significantly different from each other—indicating that dual-flip-angle Multitasking-SMS was more accurate for T2 mapping *in vivo*. T1 values were not significantly different, suggesting similar accuracy in T1. Regarding precision, the combination of SMS and dual-flip angle excitation significantly increased 3-slice SNR efficiency for both T1 and T2 vs. multitasking-SS. When traded for a 1.5 × reduction in 3-slice scan time (4.5 min to 3 min), this translated to the maintenance of T2 SNR and a 2.0 × boost in T1 SNR.

Multitasking-SMS could be a potential alternative to the conventional series of multiple T1 and T2 mapping scans in clinical studies. Conventionally, each quantitative parameter (i.e., T1/T2) is typically mapped using one breath-hold per 2D slice. As a result, 3 slices (base, mid, apex) of native T1, and T2 at diastole phase would require 6 breath holds. In a typical scenario, 3-slice T1 and T2 mapping could take ~3 min assuming a ~20 s gap between each scan for the patient to recover from the breath-hold while the technologist sets up the next scan. With an experienced MR operator, the gap can often be reduced to ~10 s and would take a total scan time of 2 min. However, shorter breath-hold recovery times may increase the likelihood of a repeat scan due to patients' difficulty in complying with breath-holds, which could then extend exam time. In our experiments, six breath-hold scans required 4–6 min. The proposed 2D CMR Multitasking-SMS removes this variability by offering a fixed 3-min scan, with the added benefits of push-button simplicity (no trigger delay times or cardiac acquisition windows to set up), free-breathing acquisition, and no ECG dependence. Further, Multitasking-SMS may have the opportunity to be extended to collect more slices in the heart with a multiband factor of 3 or more in the future.

SMS acceleration techniques have been adopted in other quantitative cardiac MRI studies (45, 46), but their data acquisition still requires breath-holding and/or ECG triggering. Multitasking-SMS is a promising free-breathing and non-ECG technique, which has tremendous potential in enhancing patient comfort, lowering technologist burden, and increasing scanner throughput. However, Multitasking-SMS also has some limitations. Qualitatively, T1 and T2 maps show some blurring, which may be due to unresolved motion or over-regularization during the reconstruction. This blurring is especially noticeable in systolic phases, although this cardiac phase is not standard for T1 and T2 analyses. A higher in-plane resolution can potentially be used to reduce the artifacts at the sacrifice of extending the scan time. Second, the reconstruction time was 2–3 h for each data acquisition, which is too long for online reconstruction in the clinic. Deep neural networks have shown promise for accelerating cardiac Multitasking reconstruction, cutting spatial factor estimation time by several orders of magnitude (47). A similar application of deep learning to the Multitasking-SMS sequence could potentially bring image reconstruction times within the clinically applicable range. Lastly, this study only evaluated Multitasking-SMS in healthy volunteers to demonstrate the feasibility of the technique. A larger study in patients is warranted.

In summary, SMS Multitasking provides co-registered T1 and T2 maps at the base, mid, and apex short-axis slices without ECG or breath-holding, all in one 3-min scan. T1 and T2 values agreed with reference measurements in a phantom and *in vivo*, and were repeatable *in vivo*. This new method improved T2 accuracy and T1 precision over the original Multitasking T1/T2 mapping method while maintaining T1 accuracy and T2 precision. The method shows potential for reducing exam time and setup time for quantitative CMR.

## Data Availability Statement

The original contributions presented in the study are included in the article/Supplementary Materials, further inquiries can be directed to the corresponding author.

## Ethics Statement

The studies involving human participants were reviewed and approved by Cedars-Sinai Medical Center. The patients/participants provided their written informed consent to participate in this study.

## Author Contributions

XM, AC, and DL: study conception and design. XM, H-LL, and FH: pulse sequence development. XM: data collection. XM and AC: analysis and interpretation of results. XM, H-LL, ZH, TC, FH, SM, FS, ZF, YX, DL, and AC: draft manuscript preparation. All authors reviewed the results and approved the final version of the manuscript.

## Funding

This work was supported by the National Institutes of Health Grant R01 EB028146.

## Conflict of Interest

FH is a full-time employee of Siemens Medical Solutions, Inc., USA. The remaining authors declare that the research was conducted in the absence of any commercial or financial relationships that could be construed as a potential conflict of interest.

## Publisher's Note

All claims expressed in this article are solely those of the authors and do not necessarily represent those of their affiliated organizations, or those of the publisher, the editors and the reviewers. Any product that may be evaluated in this article, or claim that may be made by its manufacturer, is not guaranteed or endorsed by the publisher.

## References

[1] Moon JC Messroghli DR Kellman P Piechnik SK Robson MD Ugander M. Myocardial T1 mapping and extracellular volume quantification: a Society for Cardiovascular Magnetic Resonance (SCMR) and CMR Working Group of the European Society of Cardiology consensus statement. J Cardiovasc Magn Reson. (2013) 15:1–12. 10.1186/1532-429X-15-92 24124732 PMC3854458

[2] Kvernby S Warntjes MJB Haraldsson H Carlhäll CJ Engvall J Ebbers T. Simultaneous three-dimensional myocardial T1 and T2 mapping in one breath hold with 3D-QALAS. J Cardiovasc Magn Reson. (2014) 16:1–14. 10.1186/s12968-014-0102-0 25526880 PMC4272556

[3] Akçakaya M Weingärtner S Basha TA Roujol S Bellm S Nezafat R. Joint myocardial T1 and T2 mapping using a combination of saturation recovery and T2-preparation. Magn Reson Med. (2016) 76:888–96. 10.1002/mrm.25975 26418119 PMC4811754

[4] Hamilton JI Jiang Y Chen Y Ma D Lo WC Griswold M. MR fingerprinting for rapid quantification of myocardial T1, T2, and proton spin density. Magn Reson Med. (2017) 77:1446–58. 10.1002/mrm.26216 27038043 PMC5045735

[5] Christodoulou AG Shaw JL Nguyen C Yang Q Xie Y Wang N. Magnetic resonance multitasking for motion-resolved quantitative cardiovascular imaging. Nature Biomed Eng. (2018) 2:215–26. 10.1038/s41551-018-0217-y 30237910 PMC6141200

[6] Jaubert O Cruz G Bustin A Schneider T Koken P Doneva M. Free-running cardiac magnetic resonance fingerprinting: Joint T1/T2 map and Cine imaging. Magn Reson Imaging. (2020) 68:173–82. 10.1016/j.mri.2020.02.005 32061964 PMC7677167

[7] Oh-Ici D Jeuthe S Al-Wakeel N Berger F Kuehne T Kozerke S. T1 mapping in ischaemic heart disease. Eur Heart J Cardiovasc Imaging. (2014) 15:597–602. 10.1093/ehjci/jeu024 24566951

[8] Messroghli DR Walters K Plein S Sparrow P Friedrich MG Ridgway JP. Myocardial T1 mapping: application to patients with acute and chronic myocardial infarction. Magn Reson Med. (2007) 58:34–40. 10.1002/mrm.21272 17659622

[9] Karamitsos TD Piechnik SK Banypersad SM Fontana M Ntusi NB Ferreira VM. Noncontrast T1 mapping for the diagnosis of cardiac amyloidosis. JACC Cardiovasc Imaging. (2013) 6:488–97. 10.1016/j.jcmg.2012.11.013 23498672

[10] Banypersad SM Fontana M Maestrini V Sado DM Captur G Petrie A. T1 mapping and survival in systemic light-chain amyloidosis. Eur Heart J. (2015) 36:244–51. 10.1093/eurheartj/ehu444 25411195 PMC4301598

[11] Fontana M Banypersad SM Treibel TA Maestrini V Sado DM White SK. Native T1 mapping in transthyretin amyloidosis. JACC Cardiovasc Imaging. (2014) 7:157–65. 10.1016/j.jcmg.2013.10.008 24412190

[12] Puntmann VO Carr-White G Jabbour A Yu CY Gebker R Kelle S International T1 Multicentre CMR Outcome Study. T1-mapping and outcome in nonischemic cardiomyopathy: all-cause mortality and heart failure. JACC Cardiovasc Imaging. (2016) 9:40–50. 10.1016/j.jcmg.2015.12.001 26762873

[13] Hinojar R Foote L Arroyo Ucar E Jackson T Jabbour A Yu CY. Native T1 in discrimination of acute and convalescent stages in patients with clinical diagnosis of myocarditis: a proposed diagnostic algorithm using CMR. JACC Cardiovasc Imaging. (2015) 8:37–46. 10.1016/j.jcmg.2014.07.016 25499131

[14] Thavendiranathan P Walls M Giri S Verhaert D Rajagopalan S Moore S. Improved detection of myocardial involvement in acute inflammatory cardiomyopathies using T2 mapping. Circ Cardiovasc Imaging. (2012) 5:102–10. 10.1161/CIRCIMAGING.111.967836 22038988 PMC3261300

[15] Kvernby S Rönnerfalk M Warntjes M Carlhäll CJ Nylander E Engvall J. Longitudinal changes in myocardial T1 and T2 relaxation times related to diffuse myocardial fibrosis in aortic stenosis; before and after aortic valve replacement. J Magn Reson Imaging. (2018) 48:799–807. 10.1002/jmri.25980 29473982

[16] Ugander M Bagi PS Oki AJ Chen B Hsu LY Aletras AH. Myocardial edema as detected by pre-contrast T1 and T2 CMR delineates area at risk associated with acute myocardial infarction. JACC Cardiovasc Imaging. (2012) 5:596–603. 10.1016/j.jcmg.2012.01.016 22698528 PMC3769169

[17] Kim PK Hong YJ Im DJ Suh YJ Park CH Kim JY. Myocardial T1 and T2 mapping: techniques and clinical applications. Korean J Radiol. (2017) 18:113–31. 10.3348/kjr.2017.18.1.113 28096723 PMC5240500

[18] Arai AE. Magnetic resonance imaging for area at risk, myocardial infarction, and myocardial salvage. J Cardiovasc Pharmacol Ther. (2011) 16:313–20. 10.1177/1074248411412378 21821534 PMC8690274

[19] Mordi I Carrick D Bezerra H Tzemos N. T 1 and T 2 mapping for early diagnosis of dilated non-ischaemic cardiomyopathy in middle-aged patients and differentiation from normal physiological adaptation. Eur Heart J Cardiovasc Imaging. (2016) 17:797–803. 10.1093/ehjci/jev216 26358692

[20] Taylor AJ Salerno M Dharmakumar R Jerosch-Herold M. T1 mapping: basic techniques and clinical applications. JACC Cardiovasc Imaging. (2016) 9:67–81. 10.1016/j.jcmg.2015.11.005 26762877

[21] Giri S Chung YC Merchant A Mihai G Rajagopalan S Raman SV. T2 quantification for improved detection of myocardial edema. J Cardiovasc Mag Reson. (2009) 11:1–13. 10.1186/1532-429X-11-56 20042111 PMC2809052

[22] Crouser ED Ono C Tran T He X Raman SV. Improved detection of cardiac sarcoidosis using magnetic resonance with myocardial T2 mapping. Am J Respir Crit. (2014) 189:109–12. 24381994 10.1164/rccm.201309-1668LE PMC3919128

[23] Luetkens JA Homsi R Sprinkart AM Doerner J Dabir D Kuetting DL. Incremental value of quantitative CMR including parametric mapping for the diagnosis of acute myocarditis. Eur Heart J Cardiovasc Imaging. (2016) 17:154–61. 10.1093/ehjci/jev246 26476398 PMC4882886

[24] Messroghli DR Radjenovic A Kozerke S Higgins DM Sivananthan MU Ridgway JP. Modified Look-Locker inversion recovery (MOLLI) for high-resolution T1 mapping of the heart. Magn Reson Med. (2004) 52:141–6. 10.1002/mrm.20110 15236377

[25] Piechnik SK Ferreira VM Dall'Armellina E Cochlin LE Greiser A Neubauer S Robson MD. Shortened Modified Look-Locker Inversion recovery (ShMOLLI) for clinical myocardial T1-mapping at 1.5 and 3 T within a 9 heartbeat breath-hold. J Cardiovasc Magn Reson. (2010) 12:1–11. 10.1186/1532-429X-12-69 21092095 PMC3001433

[26] Chow K Flewitt JA Green JD Pagano JJ Friedrich MG Thompson RB. Saturation recovery single-shot acquisition (SASHA) for myocardial T1 mapping. Magn Reson Med. (2014) 71:2082–95. 10.1002/mrm.24878 23881866

[27] Weingärtner S Akçakaya M Basha T Kissinger KV Goddu B Berg S. Combined saturation/inversion recovery sequences for improved evaluation of scar and diffuse fibrosis in patients with arrhythmia or heart rate variability. Magn Reson Med. (2014) 71:1024–34. 10.1002/mrm.24761 23650078

[28] Baeßler B Schaarschmidt F Stehning C Schnackenburg B Maintz D Bunck AC. Cardiac T2-mapping using a fast gradient echo spin echo sequence-first *in vitro* and *in vivo* experience. J Cardiovasc Magn Reson. (2015) 17:1–8. 10.1186/s12968-015-0177-2 26231927 PMC4522069

[29] Ehman RL McNamara MT Pallack M Hricak H Higgins CB. Magnetic resonance imaging with respiratory gating: techniques and advantages. Am J Roentgenol. (1984) 143:1175–82. 10.2214/ajr.143.6.1175 6333787

[30] Mao X Serry F Cokic I Ma S Hu Z Han F. 3D Free-Breathing, Non-ECG, T1-T2-B1+ Cine Mapping with Cardiac MR Multitasking. In: Proceedings of 24th Annual International Conference of SCMR (2021).

[31] Mao X Serry F Ma S Hu Z Han F Xie Y. 3D Whole-ventricle, Free-Breathing, Non-ECG, T1-T2-B1+ Cine Mapping with Cardiac MR Multitasking. in Proceedings of 30th Annual Meeting of ISMRM (2021). p. 690.

[32] Barth M Breuer F Koopmans PJ Norris DG Poser BA. Simultaneous multi-slice (SMS) imaging techniques. Magn Reson Med. (2016) 75:63–81. 10.1002/mrm.25897 26308571 PMC4915494

[33] Breuer FA Blaimer M Heidemann RM Mueller MF Griswold MA Jakob PM. Controlled aliasing in parallel imaging results in higher acceleration (CAIPIRINHA) for multi-slice imaging. Magn Reson Med. (2005) 53:684–91. 10.1002/mrm.20401 15723404

[34] Nezafat R Stuber M Ouwerkerk R Gharib AM Desai MY Pettigrew RI. B1-Insensitive T2 preparation for improved coronary magnetic resonance angiography at 3 T. Magn Reson Med. (2006) 55:858–64. 10.1002/mrm.20835 16538606

[35] Serry F Ma S Mao X Han F Xie Y Han H. Dual flip angle (2FA) IR-FLASH with spin history mapping for B1+-insensitive T1 mapping: Application to T1 cardiovascular magnetic resonance multitasking. Magn Reson Med. (2021) 86:3182–91. 10.1002/mrm.28935 34309072 PMC8568626

[36] Zahneisen B Poser BA Ernst T Stenger VA. Three-dimensional Fourier encoding of simultaneously excited slices: generalized acquisition and reconstruction framework. Magn Reson Med. (2014) 71:2071–81. 10.1002/mrm.24875 23878075 PMC3865111

[37] Liang Z-P. Spatiotemporal imaging with partially separable functions. Proc IEEE Int Symp Biomed Imaging. (2007) 988–91. 10.1109/ISBI.2007.357020

[38] Adluru G DiBella EV. Reordering for improved constrained reconstruction from undersampled k-space data. Int J Biomed Imaging. (2008) 2008. 10.1155/2008/341684 PMC2603268 19096715

[39] De Lathauwer L De Moor B Vandewalle J. A multilinear singular value decomposition. SIAM J Matrix Anal Appl. (2000) 21:1253–78. 10.1137/S0895479896305696

[40] Russek SE Boss M Jackson EF Jennings DL Evelhoch JL Gunter JL Sorensen AG. Characterization of NIST/ISMRM MRI system phantom. In: Proceedings of the 20th Annual Meeting of ISMRM (2012). p. 2456.

[41] Xue H Greiser A Zuehlsdorff S Jolly MP Guehring J Arai AE. Phase-sensitive inversion recovery for myocardial T1 mapping with motion correction and parametric fitting. Magn Reson Med. (2013) 69:1408–20. 10.1002/mrm.24385 22736380 PMC4138055

[42] American American Heart Association Writing Group on Myocardial Segmentation and Registration for Cardiac Imaging Cerqueira MD Weissman NJ Dilsizian V Jacobs AK Kaul S Verani MS. Standardized myocardial segmentation and nomenclature for tomographic imaging of the heart: a statement for healthcare professionals from the Cardiac Imaging Committee of the Council on Clinical Cardiology of the American Heart Association. Circulation. (2002) 105:539–42. 10.1161/hc0402.102975 11815441

[43] Weingärtner S Meßner NM Budjan J Loßnitzer D Mattler U Papavassiliu T. Myocardial T1-mapping at 3T using saturation-recovery: reference values, precision and comparison with MOLLI. J Cardiovasc Mag Reson. (2017) 18:84. 10.1186/s12968-016-0302-x 27855705 PMC5114738

[44] Von Knobelsdorff-Brenkenhoff F Prothmann M Dieringer MA Wassmuth R Greiser A Schwenke C. Myocardial T1 and T2 mapping at 3 T: reference values, influencing factors and implications. J Cardiovasc Mag Reson. (2013) 15:1–11. 10.1186/1532-429X-15-53 23777327 PMC3702448

[45] Weingärtner S Moeller S Schmitter S Auerbach E Kellman P Shenoy C. Simultaneous multislice imaging for native myocardial T1 mapping: improved spatial coverage in a single breath-hold. Magn Reson Med. (2017) 78:462–71. 10.1002/mrm.26770 28580583 PMC5509494

[46] Hamilton JI Jiang Y Ma D Chen Y Lo WC Griswold M. Simultaneous multislice cardiac magnetic resonance fingerprinting using low rank reconstruction. NMR Biomed. (2019) 32:e4041. 10.1002/nbm.4041 30561779 PMC7755311

[47] Chen Y Shaw JL Xie Y Li D Christodoulou AG. Deep learning within a priori temporal feature spaces for large-scale dynamic MR image reconstruction: application to 5-D cardiac MR multitasking. In: International Conference on MICCAI. Springer, Cham (2019). p. 495–504. 10.1007/978-3-030-32245-8_55 PMC6853633 31723946

