# Supplementary material for: Simultaneous Multi-Slice Cardiac MR Multitasking for Motion-Resolved, Non-ECG, Free-Breathing T1–T2 Mapping
Source: Front Cardiovasc Med. 2022 Mar 4;9:833257. doi: 10.3389/fcvm.2022.833257 (PMC8930916; doi:10.3389/fcvm.2022.833257)
Supplement: Supplementary file 1 [file Data_Sheet_1.docx]

**Supplementary Materials**


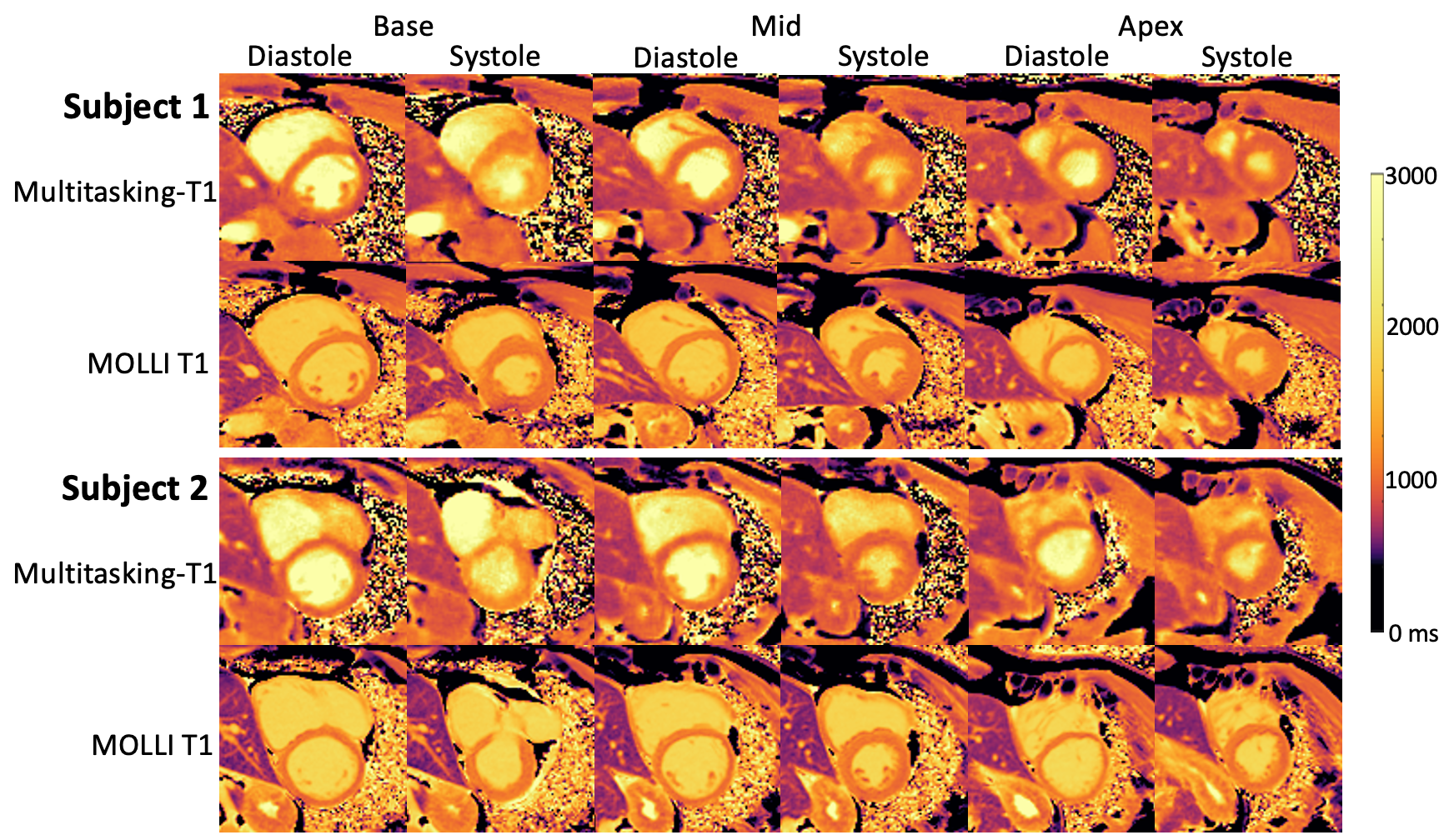


**Supplementary Figure S1** Diastolic and Systolic T1 images acquired from the proposed Multitasking-SMS method and the reference MOLLI method in two representative subjects.


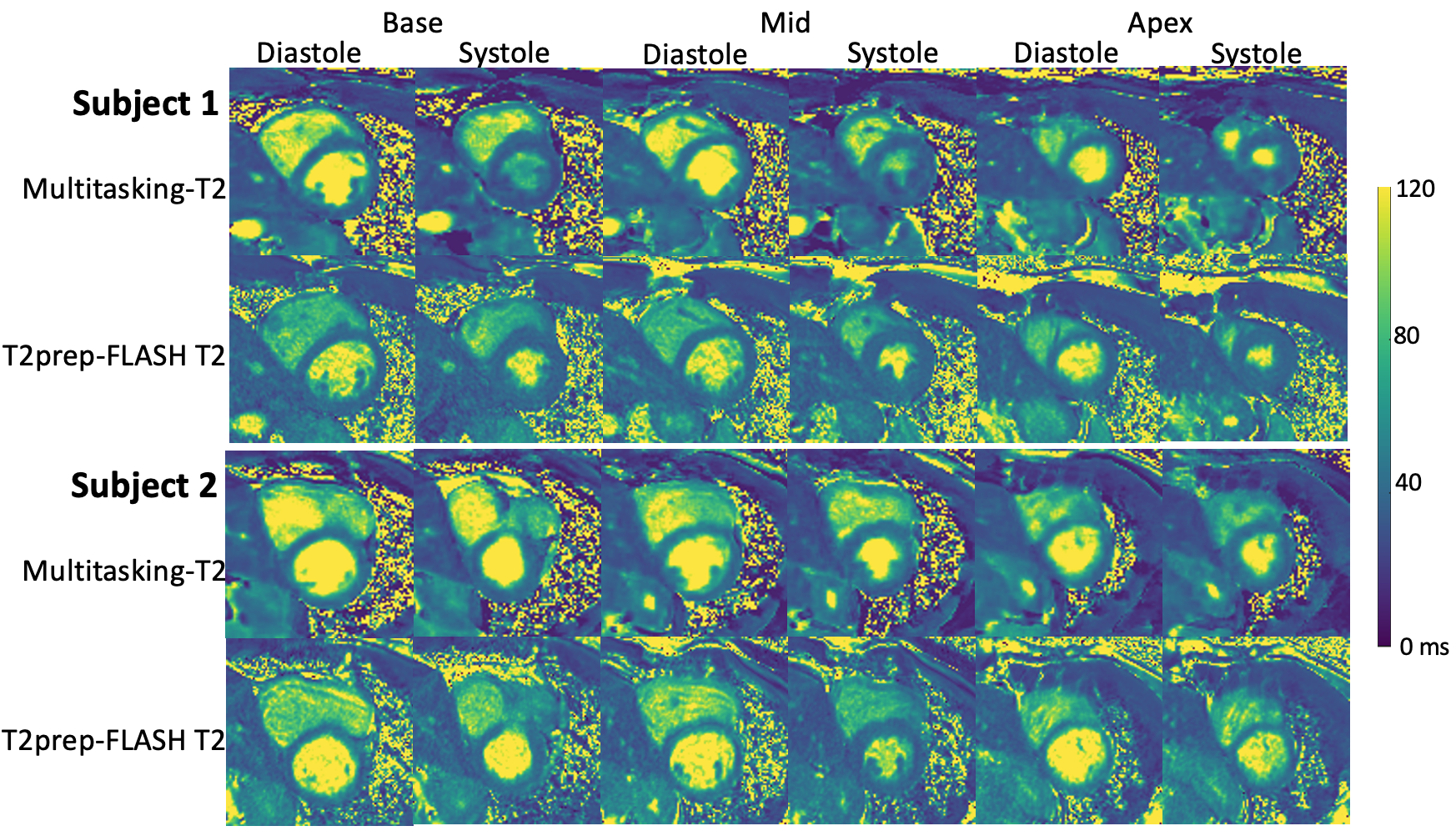


**Supplementary Figure S2** Diastolic and Systolic T2 images acquired from the proposed Multitasking-SMS method and the reference T2-prep FLASH method in two representative subjects.


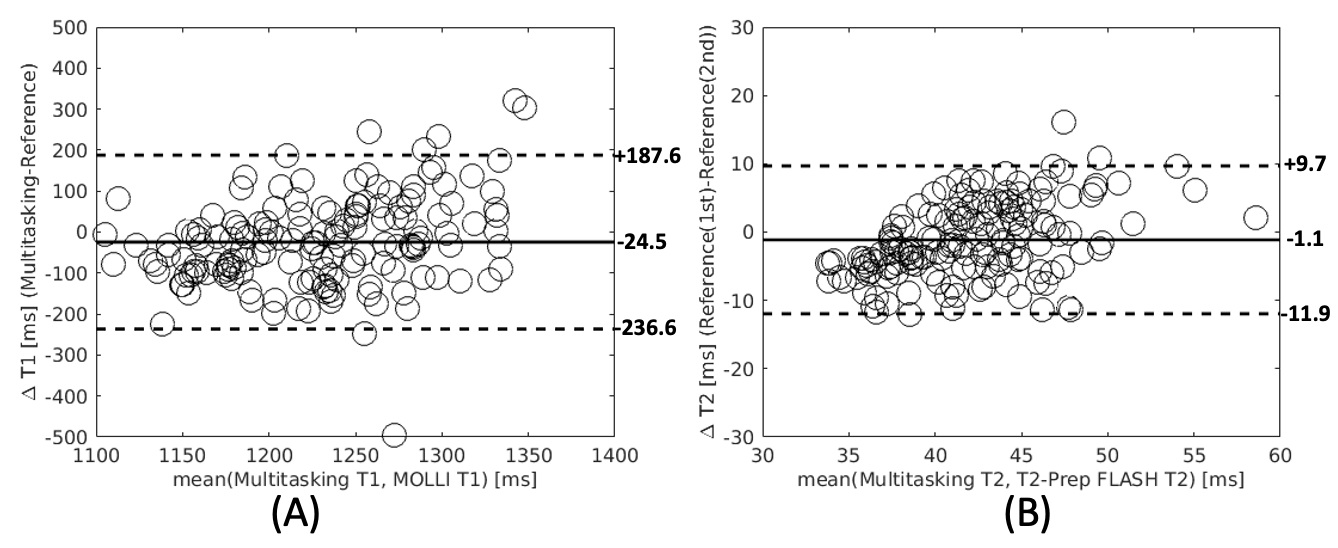


**Supplementary Figure S3** Bland-Altman plots comparing T1 (A) and T2 (B) measurements between Multitasking-SMS scans and reference scans in all 16 segments from 10 subjects. The dash lines indicate the 95% limits of agreement and the solid line indicate mean bias.


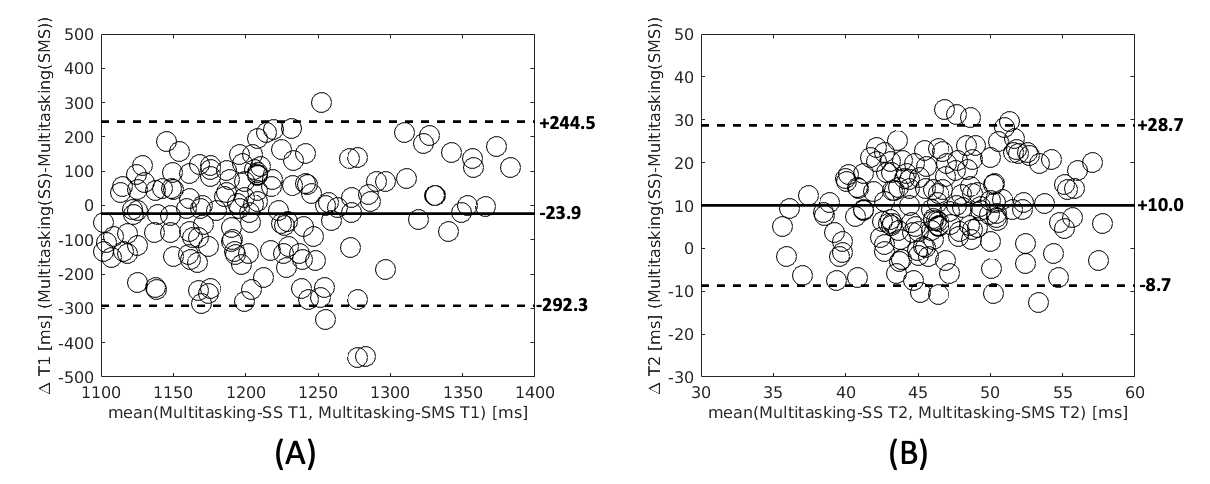


**Supplementary Figure S4** Bland-Altman plots comparing T1 (A) and T2 (B) measurements between 2D Multitasking-SS scans and Multitasking-SMS scans in all 16 segments from 10 subjects. The dash lines indicate the 95% limits of agreement and the solid line indicate mean bias.

**
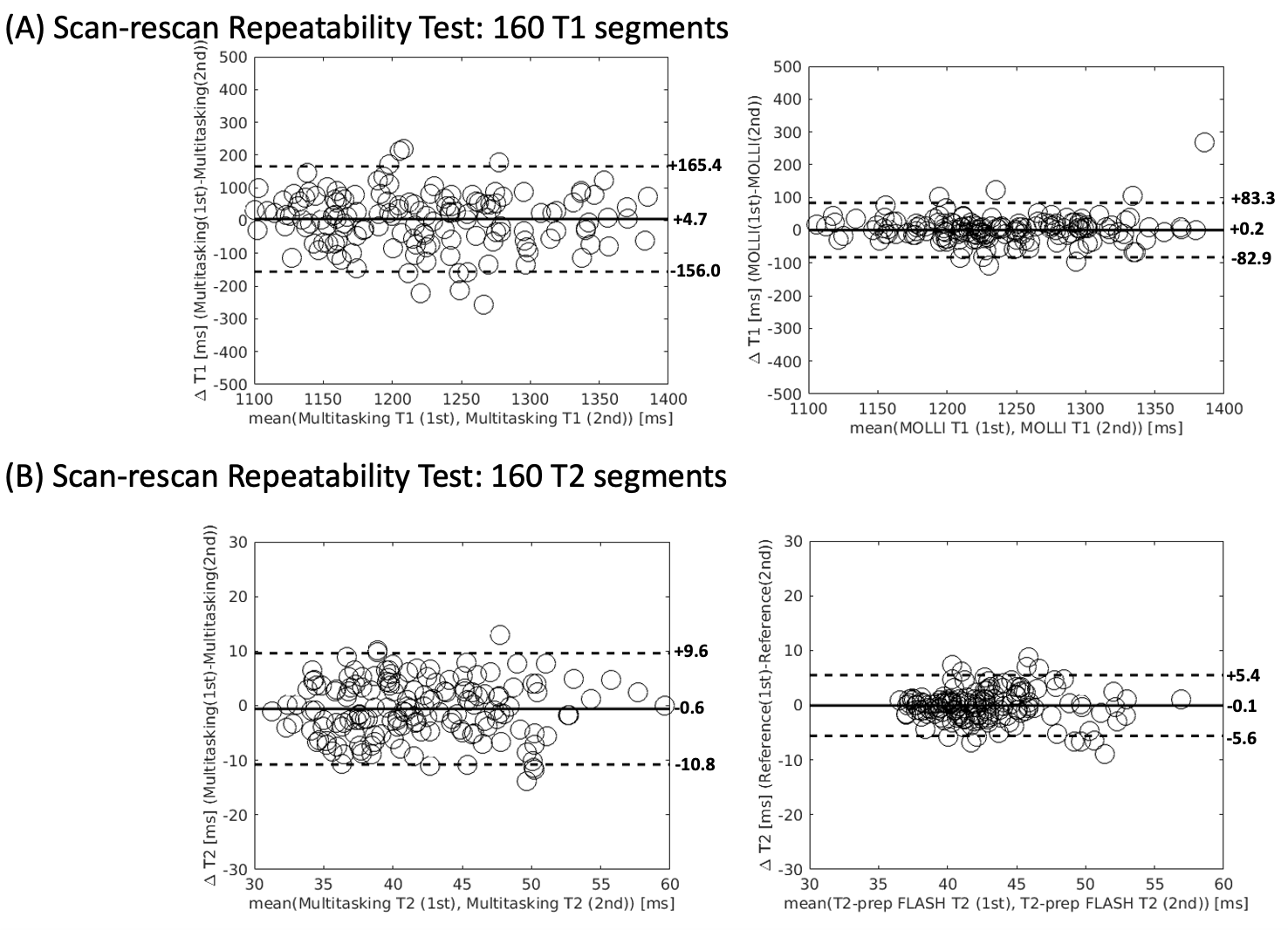
**

**Supplementary Figure S5** Bland-Altman plots comparing T1 (A) and T2 (B) measurements from 1^st^ and 2^nd^ Multitasking-SMS scans and reference scans in all 16 segments from 10 subjects. The dash lines indicate the 95% limits of agreement and the solid line indicate mean bias.


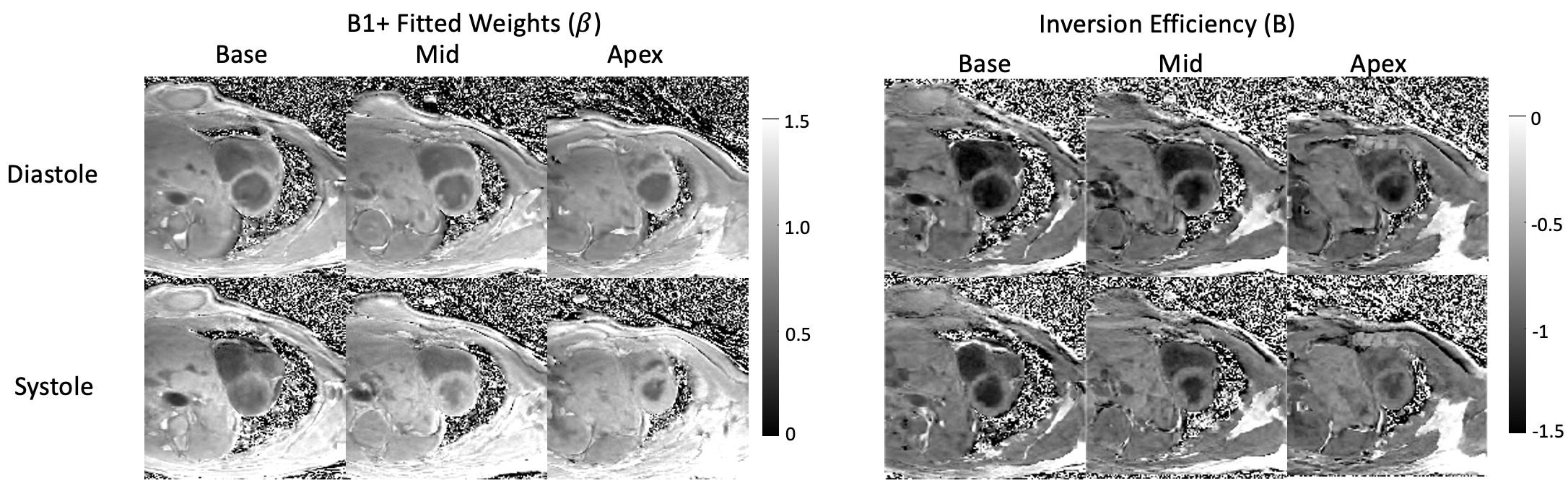


**Supplementary Figure S6** The example B1+ fitted weights ($\beta$) and inversion efficiency (B) obtained from the Multitasking-SMS fitting procedure. The B1+ field weights ($\beta$) are ranged from 0 to 1.5, the actual induced flip angle equals to the product of $\beta$ and given flip angle (3$^{\circ}$ or 10$^{\circ}$). The inversion efficiency controls the effects of inversion efficiency for the IR and T2prep-IR pulses, with “-1” means the perfect inversion.

**Supplementary Video S1** Respiratory motion extracted from the CMR Multitasking-SMS reconstruction framework from one representative subject.

**Supplementary Video S2** Cardiac motion extracted from the CMR Multitasking-SMS reconstruction framework from one representative subject.
